# Optomechanical Performances of Advanced Lightweight Mirrors Based on Additive Manufacturing

**DOI:** 10.3390/mi13081334

**Published:** 2022-08-17

**Authors:** Kai Zhang, Xiaolin Xie, Chao Wang, Ha Wang, Fang Xu, He Wang, Xin Zhang, Haijun Guan, Hemeng Qu, Jizhen Zhang

**Affiliations:** 1 Changchun Institute of Optics, Fine Mechanics and Physics, Chinese Academy of Sciences, Changchun 130033, China; 2 University of Chinese Academy of Sciences, Beijing 100049, China; 3 Smart Optics Co., Ltd., Changchun 130102, China

**Keywords:** AM, additive manufacturing, 3D printing, lightweight mirror, DfAM, design for additive manufacturing, Voronoi

## Abstract

Additive manufacturing (AM)—layer-by-layer printing—completely changes the conventional manufacturing method. The design freedom for mirrors is increased without the limits of the manufacturing process. Advanced lightweight mirrors (ALM), new-type mirrors designed using the generative method and lattice technologies, have emerged as the times require. Contrasting with conventional lightweight mirrors (CLM), the performances of ALM are drastically improved. This paper took the Voronoi mirrors as an ALM case study and introduced a design flow. In addition, a conventional honeycomb mirror was designed using the analytical method as the control. The optomechanical performances of the two were further compared through finite element analysis (FEA). Finally, ALM’s optomechanical performances outperformed CLM’s, including the area density, structural stiffness, surface stability, and quilting deflection.

## 1. Introduction

Mirrors are crucial optical elements in reflective optical systems. The lightweight rate, structural strength, and dimensional stability of mirrors directly affect the performance of optical instruments. Therefore, achieving high precision, high stability, and high reliability is vital in designing and manufacturing mirrors. A conventional lightweight mirror (CLM) is readily milled or cast to net shape and developed to the mature stage. However, the current conventional industrial design and manufacturing methods cannot meet the insatiable demand for optical systems.

**Figure 1:**
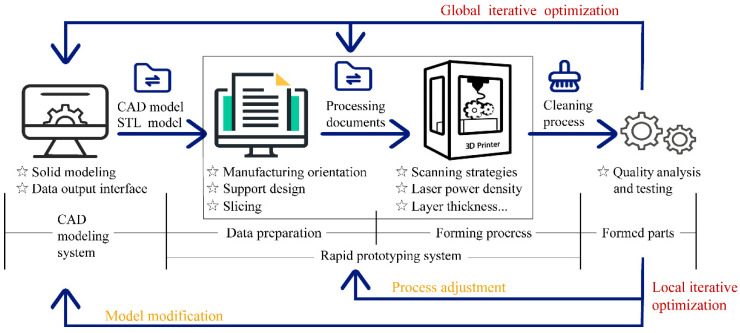
Process flow chart of AM.

Additive manufacturing (AM) from bottom to top and with layer-by-layer printing has completely changed the conventional method (Figure 1). It dramatically reduces the difficulty of manufacturing complex structures and derives a new design method, design for additive manufacturing (DfAM). The core technology of DfAM is simulation-driven optimization design technology, including creation-based design technology, topology optimization design technology, lattice design technology, parameter optimization design technology, and simulation analysis technology [1]. DfAM can redesign the whole part to maximize the advantages of AM. Advanced lightweight mirror (ALM) is a new-type mirror that was designed combined with AM and DfAM. AM and DfAM can improve the freedom and structural innovation for designing mirrors, along with a reduction in lead time.

The increase in design freedom enables more function-optimized lightweight structures and component integration. Furthermore, the increase in structural innovation makes complex surface shapes combined with complex mechanical geometries for mirrors with the characteristics of lightweight, high strength/stiffness relative to weight, and aesthetics [2]. Based on AM, researchers attempted to find excellent designs to realize the trade-off between the mechanical properties and being lightweight. Snell et al. introduced multiple lattices into the mirror design and compared the mechanical properties and manufacturability of different lattice mirrors [3]. 

Yan et al. combined the lattice mirror and assembly into a single component to realize the integrated design of the mirror [4]. Additionally, the previous work included a review of the processing technology impact on AM mirrors. Sweeney et al. studied the influence of various AM technologies and materials on the quality of mirror blanks and validated the performances of the isogrid designs [5]. Tomasz verified that selecting the correct layer thickness, laser energy density, and printing orientation had positive effects on reducing the mass of models while maintaining satisfactory mechanical properties [6,7]. Atkins et al. performed a set of comparative experiments with several technical routes and compared the surface quality of mirrors [8].

This paper took the Voronoi mirrors as our ALM case study. Due to the unique seed controllability and excellent mechanical properties, Voronoi is widely used in lightweight designs. Filling Voronoi into a mirror can decrease the unsupported area of the optical surface. Thus, it can reduce the residual surface deformation during the manufacturing process. In addition, a topology optimized mirror filled with functionally graded Voronoi can further improve the structural strength and energy absorption against environmental impacts [9,10]. 

Mici et al. proposed the design and optimization concept of a 3D-Voronoi mirror and summarized the advantages in thermal deformation and lightweight [11]. Hilpert et al. developed the 2.5D-Voronoi mirror, whose lightweight rate and mechanical properties were better than the honeycomb mirror’s [12]. While other works show successes in optomechanical performances, the design and analysis methodology for Voronoi mirrors has not been discussed in detail.

Therefore, this paper aims to develop the Voronoi ALM and contrast its design methods and performances with CLM’s. Meanwhile, the manufacturing limitations of building Voronoi ALM models are also discussed. The remainder of this paper is organized as follows. Section 2 introduces the mirror design parameters, material selection, and the design process of the mirrors. In Section 3, the optomechanical performances of several mirrors are simulated by the finite element analysis (FEA), including the area density, structural stiffness, surface stability, and quilting deflection. Finally, a discussion of the achievements closes this paper. Section 4 gives our conclusions and future work.

## 2. Materials and Methods

### 2.1. Mirror Parameters

This paper took the boundary conditions and functional requirements of the mirror for compact space optical systems as a case study. Generally, the optical design stage determines the mirror aperture and curvature radius. The mirror aperture and curvature radius are Φ120 mm and R250 mm, respectively. The sandwich mirror is an efficient, lightweight structure in bending that places most of its mass in the face and back sheets, with as little mass as possible in the shear core. As AM reduces the difficulty of manufacturing complex structures, the mirror adopted a high-performance sandwich lightweight form. 

Typically, a 6:1 aperture-to-thickness ratio is acceptable for the sandwich mirror, and thus the mirror thickness was set at 20 mm preliminarily [13]. Additionally, the maximum overall weight is an essential design requirement for space mirrors. In this paper, the expression function of area density was introduced. Table 1 provides the reference of area density for representative mirrors. The mirror aperture, curvature radiuses, lightweight form, and system weight led to an area density of less than 15 kg/m^2^. 

Space mirrors need to experience multiple states, such as the manufacturing state, detection state, assembly state, transportation state, and launch state, from manufacturing to on-orbit application. During these different conditions, the temperature and force fields change. The mirror body demands favorable stability and dynamic stiffness to minimize deformation. The integrated peripheral supporting scheme was selected, and three mounting surfaces were evenly distributed at 120° around the mirror body. Moreover, the surface shape accuracy is required to meet the visible-light imaging. Thus, the mirror also needs excellent stability. It is usually impossible to allocate a large part of the error budget to quilting. After post-polishing, the quilting deflection was limited to PV 0.03 λ (λ = 632.8 nm) ≈ 18 nm. The resulting design parameters and specifications are shown in Table 2.

### 2.2. Materials Selection

The common materials of mirrors include ceramics (SiC), glasses (glass ceramics and fused silica), and metals (aluminum and beryllium). Among them, the excellent machinability of metal leads to the ideal surface precision through ultra-precision machining technologies [14,15,16]. Metal mirrors can easily combine optical and mechanical elements to realize the integrated design and the optimal athermalization. In addition, the cost of metal mirrors is only about 50% of glass or ceramics, according to Raytheon [17]. Therefore, the metal mirror is a cost-effective and high-performance product, which has become a research highlight in recent years.

Secondly, the metal AM technology is mature. Thus, metal is an attractive material for optical mirrors because of the technological process, processing cycle, and manufacturing cost. Powder bed fusion (PBF) and directed energy deposition (DED) are two mainstream metal AM technologies in the aerospace field. Compared with DED, PBF is more suitable for manufacturing small-aperture optical mirrors with complex geometries. Among PBF technologies, selective laser melting (SLM) has a high printing accuracy with a minimum feature size of 150 μm and component density of 99.5%. In addition, the excellent surface quality, and mechanical properties of SLMed mirror blanks can reach high-quality requirements.

Aluminum alloy and titanium alloy are mainly materials for AM metal mirrors. Due to the high melting point, Ti alloy is generally used in electron beam melting (EBM) where the process cost is high. Additionally, it is unfavorable to subsequent surface processing because of its large hardness. The popular Al alloy powders include AlSi_7_Mg, AlSi_10_Mg, AlSi_12_, and AlSi_40_. The thermal expansion coefficient of AlSi_40_ can match with the NiP coating nicely, which can reduce the mirror bimetallic effect during surface modifying [18,19]. However, AlSi_40_ powder is expensive. 

Significantly, AlSi_10_Mg is the most mature powder material among them, and these printed parts have excellent microstructure and mechanical properties [20]. The surface roughness and the surface shape of the processed AlSi_10_Mg mirror can reach 5–10 nm and 0.28 λ (λ = 632.8 nm), respectively, meeting the optical imaging requirements. Therefore, the powder material for the mirror blank was AlSi_10_Mg in this paper, where the density was 2.65 g/cm^3^, the modulus of elasticity was 69 GPa, and the Poisson’s ratio was 0.33.

### 2.3. Conventional Lightweight Mirror Design

**Figure 2:**
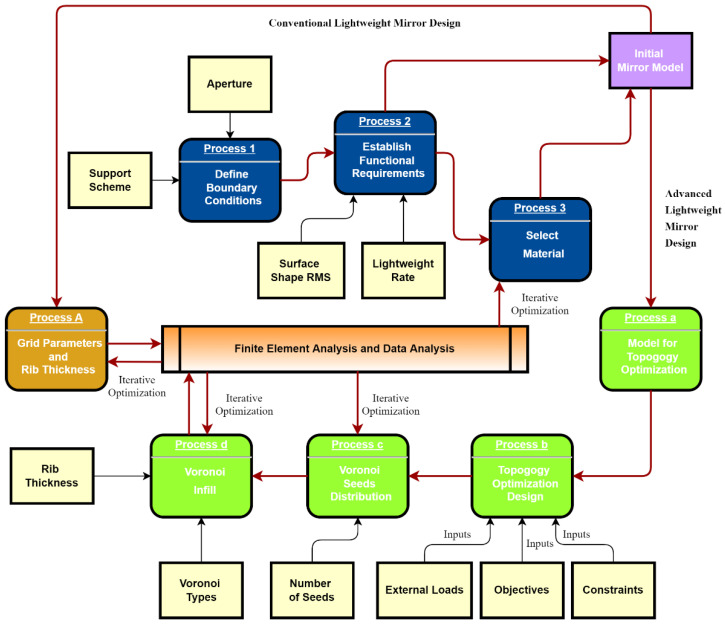
The design flow chart for CLM and ALM.

The design process of CLM and ALM is one of the main topics of this paper. Figure 2 summarizes the CLM and ALM design processes. CLM usually uses analytical and empirical methods to define rib thickness and other parameters. In contrast, ALM uses DfAM, such as topology optimization and lattice technology. This section details the design of a CLM by analytical techniques to provide a control group for the subsequent comparison.

**Figure 3:**
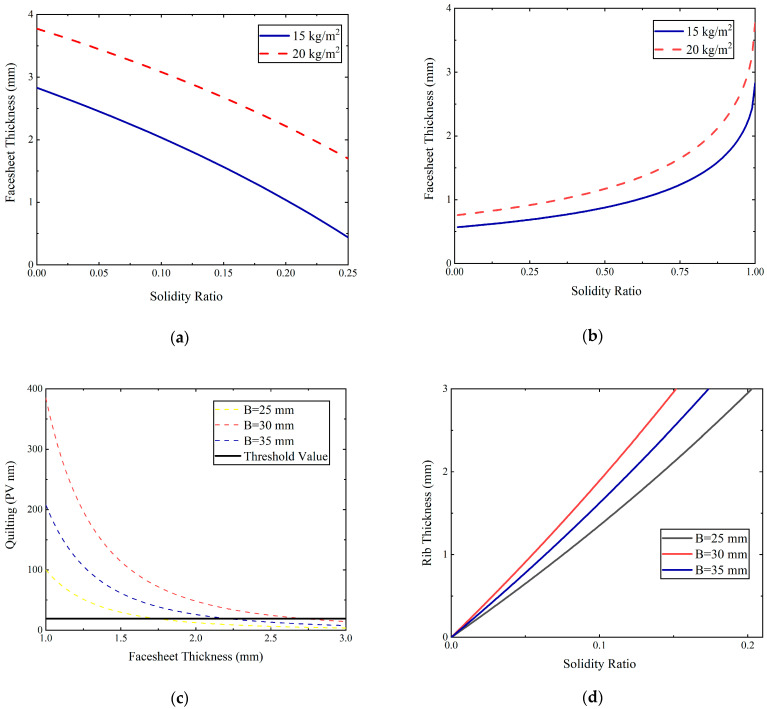
(**a**) Solidity ratio as a function of facesheet thickness. (**b**) Quilting deflection as a function of facesheet thickness. (**c**) Quilting deflection as a function of facesheet thickness. (**d**) Solidity ratio as a function of rib thickness.

The optical design stage determined an aperture of Φ120 mm and curvature radius of R250 mm. Additionally, a sandwich mirror with a thickness of 20 mm was selected as the lightweight form, as mentioned. The sandwich mirror thickness includes the facesheet, core depth, and back plate thickness. For the symmetrical sandwich mirror (the facesheet thickness is the same as the back plate thickness), the relationship between the mirror thickness is as follows:

(1) $$h=2t_{f}+h_{c},$$

where h is the thickness of the total mirror, $t_{f}$ is the facesheet or back plate thickness, and $h_{c}$ is the core depth. For determining the facesheet thickness and core depth, the area density function was introduced:

(2) $$\frac{m}{A}=\rho \left(t_{f}+\eta h_{c}\right),$$

where m/A is the area density of the mirror (the mass per unit area), ρ is the density of mirror material, and η is the solidity ratio. Substituting Equation (1) into Equation (2), the functional graph between $t_{f}$ and η is given in Figure 3a. It is a rough rule of thumb for η≤0.15. When η is 0.15, $t_{f}$ is approximately 1.8 mm. The facesheet thickness of 1.8 mm is sufficient for the manufacturing constraint. For the constant area density, Mehta studied the optimum symmetric sandwich and gave the functional relationship between $t_{f}$ and η in this case [21]:

(3) $$t_{f}=\frac{m(\sqrt{1-\frac{\eta }{2}}-\sqrt{1-\eta })}{\rho A\left\{2\left[\sqrt{1-\frac{\eta }{2}}-\sqrt{{\left(1-\eta \right)}^{3}}\right]\right\}}.$$

When η is 0.15, $t_{f}$ is 0.6 mm from Figure 3b. However, the 0.6 mm facesheet is too thin to satisfy the manufacturing requirements according to engineering experience. It is necessary to increase the thickness of the mirror facesheet to minimize quilting effects. Therefore, the thickness of the back plate and facesheet were defined as 0.6 mm and 1.8 mm, respectively. 

Quilting represents the surface deformation on the unsupported area of the mirror facesheet after polishing. Vukobratovich et al. gave the functional expression of the maximum quilting deflection [22]:

(4) $$\delta_{q}=\psi \frac{P}{E}\frac{B^{4}}{t_{f}^{3}}\left(1-\upsilon^{2}\right),$$

where $\delta_{q}$ is the maximum quilting deflection, P is the polishing pressure, E is the elastic modulus, B is the inscribed circle diameter of the pocket geometry, υ is the Poisson’s ratio for the mirror material, and ψ is a parameter depending on the pocket geometry. The pocket geometry of lightweight mirrors consists of triangular, square, or hexagonal cells. 

Among them, ψ of hexagon, 13.3 × 10^−3^, is the minimum. Thus, the hexagon pocket can get the minimum quilting deflection (Equation (4)). In addition, the equivalence of different pocket geometries is controversial. Experience with actual mirrors indicates a very weak dependence on shear core geometry [23,24]. Thus, the hexagon honeycomb with the minimum quilting deflection and the maximum lightweight rate was selected as the pocket geometry. 

The polishing pressure varied from 0.7 to 15 kPa and was normally between 1 and 2 kPa. Submitting 1.5 kPa polishing pressure for Al alloy and material parameters into Equation (4), the obtained function image between quilting deflection and facesheet thickness is shown in Figure 3c. The threshold value of quilting deflection was 18 nm, defined in Section 2.1. When B = 25 mm and $t_{f}$ = 1.8 mm, the quilting deflection $\delta_{q}$ = 17 nm is closed to the threshold value.

**Figure 4:**
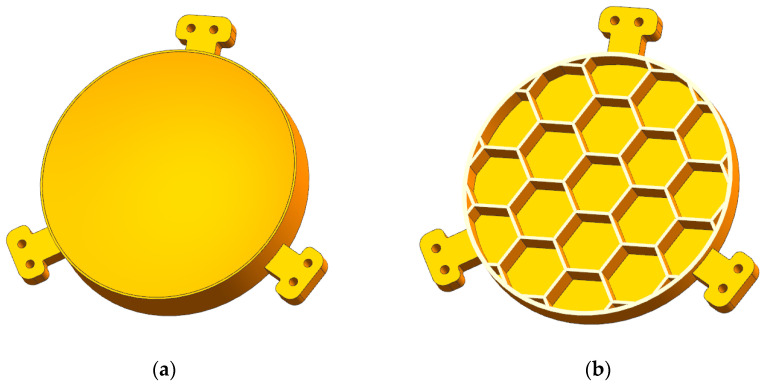
(**a**) The honeycomb mirror. (**b**) The half-cutaway view of the honeycomb mirror.

Finally, the rib thickness needs to be determined by Equation (5):

(5) $$\eta =\frac{\left(2B+t_{w}\right)t_{w}}{{\left(B+t_{w}\right)}^{2}}.$$

where $t_{w}$ is the rib thickness. Figure 3d shows the rib thickness and solidity ratio for multiple pocket geometry sizes [25]. When B = 25 mm and η = 0.15, the rib thickness $t_{w}$ is 2 mm. The final mirror model is shown in Figure 4; its weight was 182 g. The area density was 16.52 kg/m^2^, which is above the requirements of 15 kg/m^2^ by a small fraction.

### 2.4. Advanced Lightweight Mirror Design

#### 2.4.1. Voronoi

Voronoi is a group of contiguous polygons composed of lines bisected vertically, connecting the lines of two adjacent points. Since its invention, Voronoi has been widely used in various scientific fields, such as chemistry, geometry, ecology, and social economics. It is defined mathematically as [26]:

(6) $$P=\left\{p_{1},p_{2},\cdots p_{n}\right\}\in R^{m},\left(2\leq n\leq \infty \right),p_{i}\neq p_{j},i\neq j,j\in I_{n},$$

where $R^{m}$ is an m-dimensional Euclidean space and $I_{n}$ is a set of positive integers. The vertical bisectors of $p_{i}p_{j}$ lines divide the space into two halves, and $H_{i}\left(p_{i},p_{j}\right)$ represents the half-space on $p_{i}$ side, then:

(7) $$V\left(p_{i}\right)=\{x|\|x-p_{i}\|\leq \|x-p_{j}\|,\;j\neq i,\;j\in I_{n}\}=\underset{j\in I_{n}\backslash \left\{i\right\}}{\cap }H\left(p_{i},p_{j}\right),$$

where $V\left(p_{i}\right)$ is called m-dimensional Voronoi polyhedron about $p_{i}$ in $R^{m}$. The m-dimensional Voronoi diagram generated by the point set P is:

(8) $$V\left(p\right)=\left\{V\left(p_{i}\right),\cdots ,V\left(p_{n}\right)\right\}.$$

**Figure 5:**
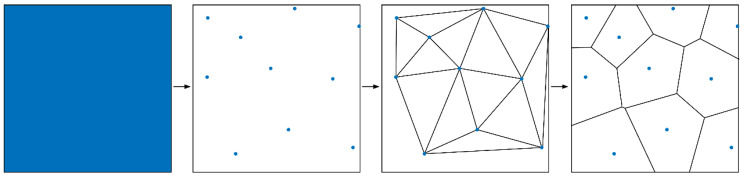
Overview for Voronoi generation.

Before the Voronoi generation, the triangle meshes formed by the connecting lines between points is Delaunay triangulation. Voronoi and Delaunay are dual graphs as shown in Figure 5.

**Figure 6:**
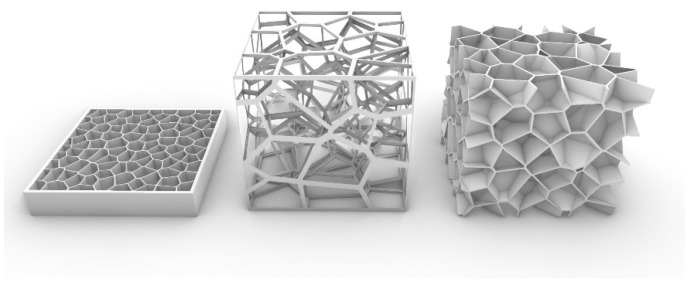
Voronoi classification: 2.5D Voronoi (left); open 3D Voronoi (middle); and closed 3D Voronoi (right).

Equations (6)–(8) are the generalized Voronoi definition. This can be expanded to the Manhattan distance Voronoi, weighted Voronoi, and high-order Voronoi, according to the distance, weight, dimensions, and order. For different dimensions, Voronoi can be divided into 2.5D and 3D Voronoi (Figure 6), which are mainly used for structural design. Based on the connectivity, 3D Voronoi can also be divided into open and closed 3D Voronoi [27]. Each structure has unique mechanical and thermal properties. Previous scholars have found the benefit of Voronoi for mirror design. However, there is little research on closed 3D Voronoi. In this paper, 2.5D-Voronoi and closed 3D-Voronoi mirrors were designed and tested as the case for the ALM study. All the 3D Voronoi below are closed 3D Voronoi.

#### 2.4.2. Topological Optimization

The ALM design adopted functionally graded structures as shown in Figure 2. The optimal material distribution of the mirror design area can be obtained by topological optimization under constraints. Then, taking the topology optimization results as a reference, Voronoi’s seed distribution was interfered with to achieve a practical design. This paper used the variable density methods solid isotropic material penalty (SIMP) to optimize mirrors. 

SIMP is derived from the idea of microstructure equivalence. It takes the relative density of elements as the design variable and realizes the topological structure through the spatial configuration change of density from 0 to 1 [28]. Compared with other methods, SIMP has good stability and topology change capacity. Additionally, the evolution of density is performed on the finite element mesh, which is easy to realize through computer programs.

The wavefront root mean square (RMS) is a common method to evaluate the mirror surface shape. It is defined as the RMS of the distance between the deformed node and the fitting surface:

(9) $$\mathrm{RMS}=\sqrt{\sum_{i=1}^{n}\frac{1}{n}{\left(x_{i}-\bar{x}\right)}^{2}}.$$

where $x_{i}$ is the distance from the i-th node to the fitting surface deformation, and $\bar{x}$ is the average distance between all nodes and the fitting surface. The existing commercial software for topological optimization cannot use RMS as the optimization objective [29]. The structural compliance was selected to replace the wavefront RMS of the optical surface. For minimizing the RMS, the minimum compliance of the structure was selected as the objective function to minimize the displacement of the surface nodes. The optimization model is as follows:

(10) $$\mathrm{Find}\;\;X=\left(\rho_{1},\rho_{2},\cdots \rho_{N}\right),$$

and the optimization objective:

(11) $$\mathrm{Minimize}\;C\left(x\right)=U^{T}KU,$$

subject to:

(12) KU=F,

(13) $$V_{min}\left(x\right)=\sum_{i=1}^{n}\rho_{i}v_{i}\;\left(i=1,2,\cdots ,N\right),$$

(14) $$0\leq \rho_{i}\leq 1.$$

In this model, $\rho_{i}$ is the relative density. U is the global displacement vector, and K is the global stiffness matrix. F is the global load vector. V is the total volume of the structure, and $v_{i}$ is the unit volume. N is the number of unit variables. 

**Figure 7:**
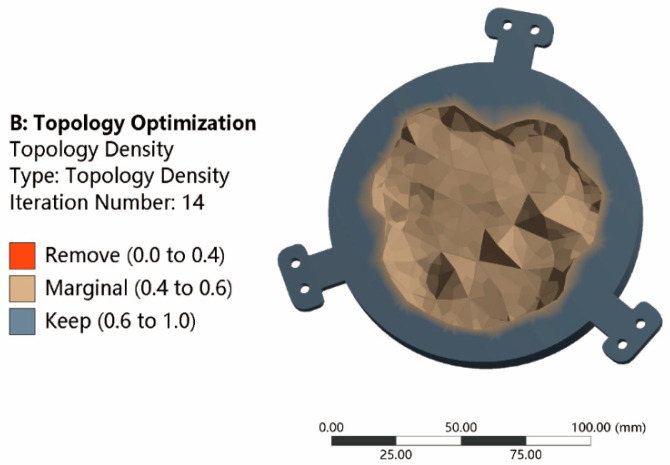
The results of the topological optimization (back of the mirror).

A 30% residual volume was set as the response constraint. After 14 iterations, the optimization curve gradually converged to a stable value. Additionally, the topology optimization result is shown in Figure 7. The relative density of materials can determine the structural configuration. During the optimization process of the variable density method, the elements with a relative density less than 0.4 were removed, and the elements with a relative density greater than 0.6 were kept. The distribution design of marginal area with relative density between 0.4–0.6 was critical, which will be discussed later.

#### 2.4.3. 2.5D-Voronoi Mirror

The 2.5D-Voronoi mirror can be understood as the ribs distributed as a 2D Voronoi diagram inside the mirror. The above topology optimization results gave the optimal distribution of the relative density of the mirror materials. We determined the distribution of Voronoi seeds according to the topology optimization results to realize the functionally graded structures. The internal design area of the mirror was divided into a dense and a sparse area. The diameter of the sparse area was Φ90 mm.

**Figure 8:**
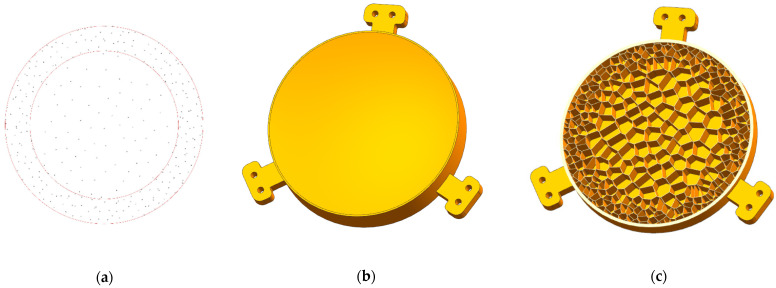
(**a**) Seed distribution of 2.5D-Voronoi mirror; (**b**) 2.5D-Voronoi mirror; and (**c**) half-cutaway view of 2.5D-Voronoi mirror.

According to the engineering experience, the seed number was set as 150 preliminarily in the sparse area. Additionally, seed number of the dense area was designated as 200. Figure 8a shows the distribution of the seeds. For SLM, the minimum wall thickness of the unsupported rib is limited to 0.3 mm to avoid deformation [30]. Based on the manufacturing constraints, the rib thickness was 0.6 mm. In addition, the transitional elements of the marginal area were processed with smooth interconnectivity. The final model of the 2.5D-Voronoi mirror is shown in Figure 8b,c. The weight of the 2.5D-Voronoi mirror was 156 g, which was lighter than the honeycomb mirror by 26 g under the same boundary conditions. Additionally, the area density of 14.16 kg/m^2^ achieved the requirement.

#### 2.4.4. 3D-Voronoi Mirror

3D-Voronoi mirror frees up the design space in the Z-direction. Its design freedom is significantly higher than the 2.5D-Voronoi mirror. Moreover, 3D Voronoi can provide more homogenized and denser support for the mirror surface than 2.5D Voronoi. The design area for the 3D-Voronoi mirror was divided into the surface support area (under the mirror surface 5 mm, diameter Φ120 mm), the dense external area (15 mm above the back plate, diameter Φ90 mm–Φ120 mm), and the sparse internal area (15 mm above the back plate, diameter Φ90 mm), according to the topology optimization result. 

**Figure 9:**
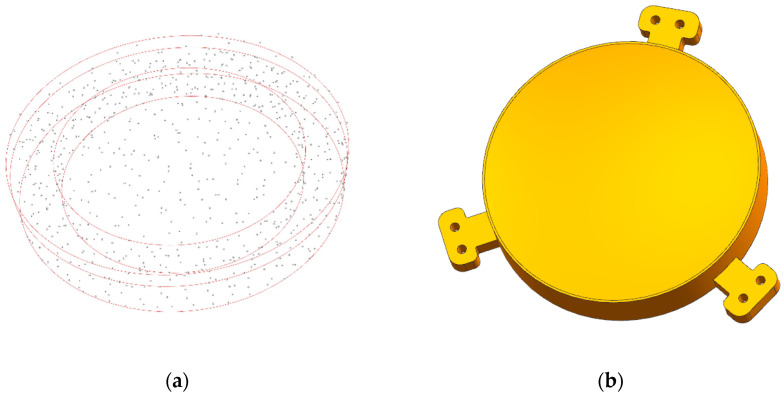

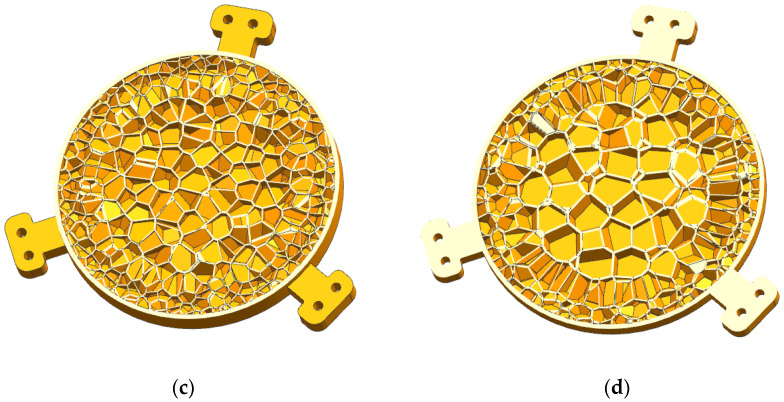
(**a**) Seed distribution of 3D-Voronoi mirror; (**b**) 3D-Voronoi mirror; (**c**) half-cutaway view of 3D-Voronoi mirror; and (**d**) 70%-cutaway view of 3D-Voronoi mirror.

The seeds in the surface support and dense external areas were more than those in the sparse internal area. The number of seeds in the surface support and dense external areas was 200, yet the sparse internal area was 100. The seed distribution is shown in Figure 9a. With each seed as the center, the polyhedrons are indented in proportion to generate the volume models. Due to the limitations of the minimum wall thickness, 3D-Voronoi cell sizes were controlled within a certain range. 

The maximum overhang angle is within 30° during the printing of aluminum alloy [30]. Therefore, the printing direction and support design are worthy of attention for the complex 3D-Voronoi. The subsequent iterative optimization of design and manufacturing is even required (Figure 1). The final 3D-Voronoi mirror model is shown in Figure 9b–d. Its weight was 163 g, between the honeycomb mirror and the 2.5D-Voronoi mirror. The area density of 14.8 kg/m^2^ approached the requirement of 15 kg/m^2^.

## 3. Results and Discussion

In this section, FEA was performed to evaluate the mirror performances. During the analysis, the calculation time mainly depends on the finite element mesh size and quantity. However, finely meshing the complex Voronoi structures is necessary to retain the complete structural features, especially 3D Voronoi. The numerous and small Voronoi meshes lead to lots of time to pre-process and solve in simulation. Thus, we used the equivalent model to simulate the modal frequency, surface deformation, and quilting deflection for the analysis efficiency and accuracy.

### 3.1. Modal Analysis

Modal analysis is to solve the natural frequency and vibration mode of the mirror. The results are critical indicators for evaluating the dynamic stiffness of mirrors. The resonance between optomechanical structures can be avoided after modal analysis. Modal analysis can also obtain the stiffness characteristics and serve as the input conditions for other studies, such as harmonic response analysis and random vibration analysis [31]. These analyses play a significant role in the evaluation of structural design schemes. The modal frequency is negatively correlated with the mass because both the final Voronoi mirrors are lighter than the honeycomb mirror. We adjusted the models slightly for accurate modal analysis to realize the equal group.

**Figure 10:**
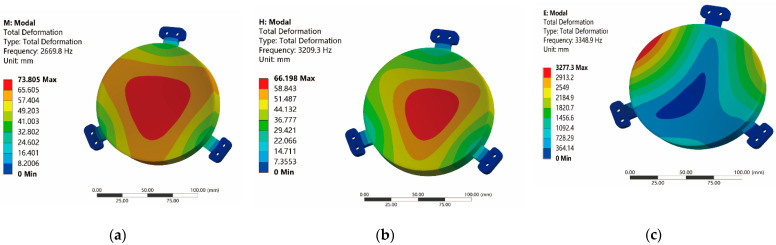
Results of first-order modal: (**a**) honeycomb mirror; (**b**) 2.5D-Voronoi mirror; and (**c**) 3D-Voronoi mirror.

The same clamping fixed the mirror models. Additionally, the modal analysis of the mirror bodies was performed, constraining all the free degrees of the mounting surfaces. The first-order modal results of mirrors are shown in Figure 10. The first-order frequency of 2669 Hz for the honeycomb mirror failed to meet the design requirements. However, the first-order modal frequencies of 2.5D-Voronoi and 3D-Voronoi mirrors were increased by 20% (3200 Hz) and 25% (3348 Hz), respectively, compared with the honeycomb mirror. 

**Figure 11:**
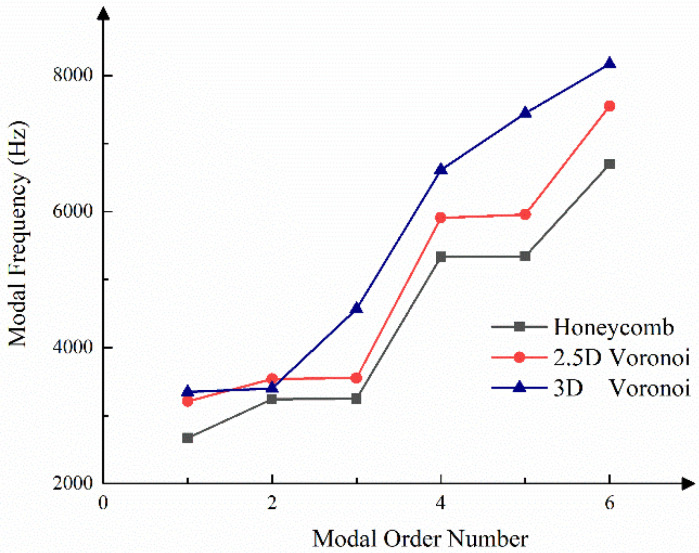
The front six modes and the corresponding frequency of the mirrors.

Both first-order modal frequencies of the 2.5D-Voronoi and 3D-Voronoi mirrors exceeded the requirements. While the models of Voronoi mirrors were modified, the practical modal frequency was higher than the analytical results. Figure 11 gives the modal analysis’s front six modes and the corresponding frequency. Each modal frequency of the 3D-Voronoi mirror was almost the largest. Thus, the 3D-Voronoi mirror had the maximum structural strength.

### 3.2. Forced Displacement Analysis

First-order modal frequency is a direct response to structural stiffness. Nevertheless, the higher the structural stiffness, the greater the influence of external stress. Due to the integrated design of the mounting structure and mirrors, the screw tightening force can be transferred to the optical surface during integration and alignment, which affects the surface shape accuracy. Therefore, the forced displacement analysis was performed to evaluate the comprehensive optomechanical performance.

**Figure 12:**
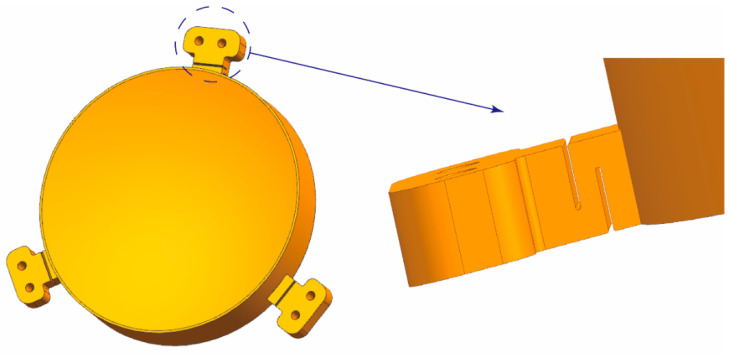
The flexible supporting structure.

The flexible structure was designed according to the surface shape accuracy requirements (Figure 12) and realized by the wire-electrode cutting technology after AM. It can isolate the influence of screw tightening force on the optical surface and absorb specific vibrations to realize the effect of unloading stress [11]. The deformations in multiple directions were considered to improve the mirror adaptability during the flexible structure design. At the same time, the distance between the mounting surface and the mirror should be increased as much as possible to extend the propagation distance of the tightening force and attenuate transmission.

**Figure 13:**
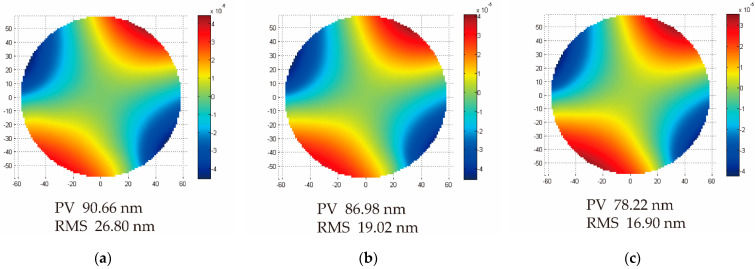
Surface shape fitting nephograms after the forced displacement analysis: (**a**) honeycomb mirror; (**b**) 2.5D−Voronoi mirror; and (**c**) 3D−Voronoi mirror.

Two mounting structures were fixed during the analysis, and the 3 μm displacement in the Z-direction was applied to the remaining mounting structure. Figure 13 shows the fitting results of the deformation of the mirror surface. Nephograms showed that the deformations of optical surfaces were all astigmatism with small differences. The deformation of the honeycomb mirror was the largest—RMS 26.80 nm (1/25 λ, λ = 632.8 nm). The 3D-Voronoi mirror was superior to the honeycomb mirror—RMS 16.90 nm (1/39 λ, λ = 632.8 nm). The 2.5D-Voronoi mirror was between the two—RMS 19.02 nm (1/33 λ, λ = 632.8 nm). Therefore, the 3D-Voronoi mirror demonstrated excellent overall structural stiffness and anti-deformation capabilities on the optical surface. 

### 3.3. Quilting Analysis

Quilting, also known as the “print-through,” is the residual surface deformations caused by the unsupported areas in the manufacturing process. These unsupported areas are located on the unit between the rib structures, reducing the optical surface’s stiffness. Additionally, the deformation is permanent in the form of upward expansion or dimple. Due to the rib structures, the pressure between the polishing tool and the surface changes. The area with rib support has more excellent bending resistance to the tool compared with the hole center. 

**Figure 14:**
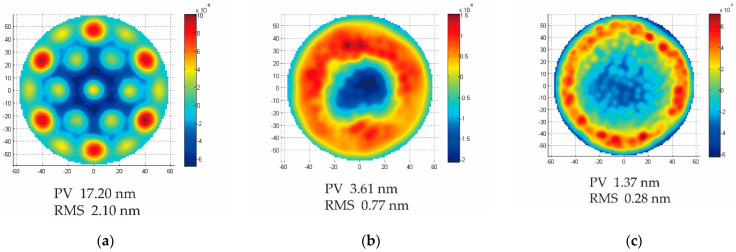
Surface shape fitting nephograms after the post-polishing analysis: (**a**) honeycomb mirror; (**b**) 2.5D-Voronoi mirror; and (**c**) 3D-Voronoi mirror.

Moreover, quilting is an internal trade-off for the lightweight mirror design. Since the mirror surface is processed by the small tool polishing and other shape modification, the quilting analysis is necessary. Section 2.3 gave the equation about the maximum quilting deflection. During the quilting analysis, the polishing pressure was set at 1.5 kPa. Figure 14 shows the fitting nephograms of the optical surfaces. Among them, the quilting deflection of the honeycomb mirror was PV 17.2 nm, and 2.5D-Voronoi and 3D-Voronoi reduced the quilting deflection significantly for mirrors. The quilting reduced 79% for the 2.5D-Voronoi mirror and 92% for the 3D-Voronoi mirror compared with the honeycomb mirror.

Interestingly, there were also differences in the surface errors of the frequency range. It can be seen from the nephograms that the quilting of the honeycomb mirror focused on the mid-spatial frequency roughness (MSFR). 2.5D-Voronoi mirror and 3D-Voronoi mirror focused on the low-spatial frequency roughness (LSFR) and high-spatial frequency roughness (HSFR). MSFR can cause small-angle scattering, thus, reducing the contrast of the image. It is more difficult to remove than LSFR and HSFR, considering the processing accuracy. MSFR needs to be detected by laser-scanning microscope or white-light interferometer. 

## 4. Conclusions and Future Work

This paper summarized the design methodologies of CLM and ALM. A conventional honeycomb mirror was designed based on the analytical method. Novel Voronoi mirrors were designed via topological optimization to realize functionally graded structures. Finally, the comprehensive optomechanical performances of the mirrors were evaluated through FEA. The mirror performances are shown in Table 3.

All the analysis results of the honeycomb mirror were close to the design requirements. The Voronoi mirrors outperformed the overall honeycomb mirror performances. First-order modal frequencies of 2.5D-Voronoi and 3D-Voronoi mirrors improved by 20% and 25%, respectively. In addition, the surface deformations from forced displacement of 2.5D-Voronoi and 3D-Voronoi mirrors were enhanced by 23% and 34%, respectively. This indicates the high dynamic stiffness and stability of Voronoi mirrors. 

As Voronoi provides homogenized and dense support for the mirror surface, the quilting deflection of Voronoi mirrors reduces widely, which is beneficial for subsequent processing. In particular, the quilting deflection of the 3D-Voronoi mirror increased remarkably by 92% from the honeycomb mirror. In summary, these results suggest that the optomechanical performances of Voronoi mirrors are superior. This provides a novel methodology for designing lightweight mirrors.

In the future, the optomechanical performances will be verified based on the combination of manufacturing technology and experiments. Expansions to thermal and other performances for the Voronoi mirrors should be considered. 

## Figures and Tables

**Table 1:** Area density for representative mirrors.

Mirror	Diameter (m)	Type	Material	Area Density (kg/m^2^)
MMT ^1^	1.8	Sandwich	Fused silica	223
HST ^2^	2.4	Open back	ULE	160
Kepler	1.45	Sandwich	ULE	50
Spitzer	0.85	Single arch	Beryllium	28
ESO VLT	1.14	Open back	Beryllium	39
JWST (segment) ^3^	1.52	Open back	Beryllium	14

^1^ MMT, Smithsonian Astrophysical Observatory Multiple Mirror Telescope; ^2^ HST, Hubble Space Telescope; and ^3^ JWST, James Webb Space Telescope.

**Table 2:** Design requirements of the mirror.

Parameter	Specification
Aperture	120 mm
Curvature radiuses	250 mm
Thickness	20 mm
Area density	15 kg/m^2^
First-order modal frequency	2800 Hz
Surface shape accuracy	RMS 15 nm
Quilting deflection	PV 18 nm

**Table 3:** Summary of the mirror performances.

	Honeycomb Mirror	2.5D-Voronoi Mirror	3D-Voronoi Mirror
Area Density	16.52 kg/m^2^	14.16 kg/m^2^	14.8 kg/m^2^
First-order Modal Frequency	2669 Hz	3209 Hz	3348 Hz
Deformation from Forced Displacement (RMS)	26.80 nm	19.02 nm	16.90 nm
Quilting Deflection (PV)	17.20 nm	3.61 nm	1.37 nm

## Data Availability

Not applicable.
